# Nationwide Surveillance in Uterine Cancer: Survival Analysis and the Importance of Birth Cohort: 30-Year Population-Based Registry in Taiwan

**DOI:** 10.1371/journal.pone.0051372

**Published:** 2012-12-10

**Authors:** Chia-Yen Huang, Chi-An Chen, Yu-Li Chen, Chun-Ju Chiang, Tsui-Hsia Hsu, Ming-Chieh Lin, Mei-Shu Lai, Chien-Jen Chen, San-Lin You, Wen-Fang Cheng

**Affiliations:** 1 Gynecologic Cancer Center, Cathay General Hospital, Taipei, Taiwan; 2 Department of Obstetrics and Gynecology, National Taiwan University, Taipei, Taiwan; 3 Graduate Institute of Epidemiology, College of Public Health, National Taiwan University, Taipei, Taiwan; 4 Bureau of Health Promotion, Department of Health, Executive Yuan, Taipei, Taiwan; 5 Department of Pathology, National Taiwan University, Taipei, Taiwan; 6 Institute of Life Sciences, School of Public Health, National Defense Medical Center, Taipei, Taiwan; 7 Genomics Research Center, Academia Sinica, Taipei, Taiwan; 8 Graduate Institute of Oncology, National Taiwan University, Taipei, Taiwan; 9 Graduate Institute of Clinical Medicine, College of Medicine, National Taiwan University, Taipei, Taiwan; Centers for Disease Control and Prevention, United States of America

## Abstract

**Introduction:**

Uterine cancer was the most rapidly increasing malignancy and the second most common gynecologic malignancy in Taiwan.

**Methods:**

We analyzed the secular trend of uterine cancer incidence and compare the survival of women with uterine carcinomas and uterine sarcomas in Taiwan. Data on women diagnosed with uterine cancer between 1979 and 2008 were obtained from the Taiwan cancer registry. Survival data were analyzed by using Kaplan-Meier and Cox proportional hazards regression methods.

**Results:**

Records of 11,558 women with uterine carcinomas and 1,226 women with uterine sarcomas were analyzed. The age-adjusted incidence rate of endometrioid adenocarcinoma increased from 0.83 per 100,000 women per year between 1979 and 1983 to 7.50 per 100,000 women per year between 2004 and 2008. The 5-year survival rate of women with endometrioid adenocarcinoma (83.2%) was higher than that for women with clear cell carcinoma (58.3%), serous carcinoma (54.4%), and carcinosarcoma (35.2%) (*p*<0.0001, log-rank test). The 5-year survival rates of women with low grade endometrial stromal sarcoma, endometrial stromal sarcoma, leiomyosarcoma (LMS), and adenosarcoma were 97.5%, 73.5%, 60.1%, and 77.2%, respectively (*p*<0.0001, log rank test). The histologic type of endometrioid adenocarcinoma, young age, and treatment period after 2000 were independent, favorable prognostic factors in women with uterine carcinomas by multivariate analysis. The histologic type of LMS, old age, and treatment period after 2000 were independent, poor prognostic factors in women with uterine sarcomas by multivariate analysis.

**Conclusions:**

An increase over time in the number of patients with endometrioid adenocarcinomas was noted in this 30-year, nationwide, population-based study. Histologic type, age and treatment period were survival factors for uterine cancers. A more comprehensive assessment of uterine cancers and patient care should be undertaken on this increasingly common type of cancer.

## Introduction

Uterine cancer is the most common gynecologic cancer in the United States, with 47,130 new cases projected in 2012 [1]. In Taiwan, it is the second most common gynecologic cancer, with 1,424 newly diagnosed cases of uterine cancer in 2009 [2]. The incidence of uterine cancer has remained stable in the past 20 years in the United States [3]. However, the age-adjusted incidence of uterine cancer (all females of any age) dramatically increased from 1979 to 2007 in Taiwan (0.99 per 100,000 women per year in 1979 and 8.26 per 100,000 women per year in 2007) [2], and uterine cancer was the most rapidly increasing malignancy in Taiwanese women. Despite its importance, no published population data have focused on uterine cancer in Taiwan.

Uterine cancers are divided into two major categories; uterine carcinomas and uterine sarcomas. Uterine carcinomas account for the majority of cases of uterine cancer, while uterine sarcomas are rare and only account for approximately 4.2% of all corpus uteri malignancies [4]. Uterine carcinomas are categorized as type I and type II carcinomas based on the pathogenesis of disease and clinical behavior of the patients [5]. Endometrioid adenocarcinoma, regarded as type I carcinoma, accounts for about 80% of uterine carcinomas [6]. Papillary serous carcinomas, clear cell carcinomas, and carcinosarcomas, regarded as type II carcinoma, account for less than 10% of uterine carcinomas [4], [7]–[9]. Because of the rarity of papillary serous carcinomas, clear cell carcinomas, and carcinosarcomas, only a few population-based, follow-up studies on the outcomes of these types of uterine cancers have been reported [4], [10].

Uterine sarcomas are generally categorized into endometrial stromal sarcoma (ESS), leiomyosarcoma (LMS), and adenosarcoma. Because of the rarity of uterine sarcomas, there are also only a limited number of published reports on the outcomes [11], and most of the outcome studies on uterine sarcomas have been based on small retrospective series from a single institution, which lack power to make significant conclusions [12], [13].

We undertook this nationwide, population-based study on the outcomes of 11,502 patients with uterine cancers to identify changes in the incidence of uterine cancers, prognostic factors of uterine cancers, and the influence of different birth cohorts on uterine cancer.

## Methods

### Data Sources from the National Cancer Registry System

There are 23 million people in Taiwan, with a low migration rate, convenient transportation, modest difference in socioeconomic development between urban and rural areas, and an excellent health-care system even in remote areas. Nearly all cancer patients in Taiwan are diagnosed and treated in hospitals. The Department of Health in Taiwan launched the National Cancer Registry system in 1979 to collect information on all cancer cases from hospitals with 50 or more beds based on the *International Classification of Diseases for Oncology*. The registry is considered to be complete and accurate, with the percentage of cases based on death certificates only (DCO) as low as 1.5%. The DCO percentages for gynecologic cancers including cervical, uterine, and ovarian cancers were all lower than 0.5% in 2007. Due to the regulation of National Health Insurance of Taiwanese government, if a patient’s ailment is diagnosed as a “catastrophic illness” (such as malignant neoplasms, end-stage renal disease, systemic lupus erythematosus, and etc.) under Department of Health guidelines, the patient can submit related information and apply for a catastrophic illness certificate. Patients with the catastrophic illness certification who get care for the illness or related conditions within the certificate’s validity period do not need to pay the copayment of outpatient or inpatient care. However, the diagnosis of malignant neoplasms should be made by morphological verification. So the percentages of morphological verification of cervical, uterine, and ovarian cancers were all greater than 99%. The research protocol was approved by the Institutional Review Board of National Taiwan University Hospital.

### Ascertainment of Incidence and Cases of Death

Information on uterine cancer cases diagnosed between January 1, 1979, and December 31, 2008, was retrieved from the National Cancer Registry of Taiwan. The population data was obtained from National Household Registration Profiles. In addition, the incidence of female nasopharyngeal carcinoma over the same period of time was used as internal references to avoid the potential bias due to incomplete cancer registry data. The diagnosis of nasopharyngeal carcinoma is usually not difficult, and there was no reason to expect any change in incidence during the study period. Registry data included date of birth, date of diagnosis, anatomical site of the tumor, histological diagnosis, and treatment. The yearly histological confirmation rate ranged from 99% to 100% for all registered cancers. Women affected by uterine cancer and found to have died due to uterine cancer (International Classification of Diseases 9th edition code 182) on death certificates were defined as death cases. The Taiwanese government launched the National Health Insurance (NHI) program in 1994. After 1994, almost all patients with cancers including uterine cancers were treated and covered by the NHI. Therefore, we took the patients treated between 1995 and 1999 as reference data, and compared their outcomes with those treated before and after this period to investigate whether there were any differences between different treatment periods. Because the registration of death certificates was not comprehensive before 1990, so we only used 11,502 cases of uterine cancers diagnosed between January 1, 1990, and December 31, 2008 for survival analysis. For analyses, 10-year groupings were used to evaluate the influence of age; for calendar years, data were grouped into six 5-year intervals (from 1979 to 2008). The year of birth was calculated from the year of diagnosis and age at diagnosis; fourteen 5-year birth cohorts (from 1909 to 1978) were then analyzed.

### Classification of Histopathological Subtypes of Uterine Cancers

Women diagnosed with a primary cancer of the uterus (ICD-O3 C54.0–C54.3, C54.8–C54.9, C55.9) with valid histological codes from 8000–8991, between 1979 and 2008 were eligible for this study. The data before 2002 was registered according to ICD-O-FT (ICD-O-FT 182) and was shifted to ICD-O3. On the basis of widely accepted guidelines [14], [15]), the histopathological subtypes of uterine cancers were classified according to the World Health Organization Classification of Tumors (Tumors of Breast and Genital Organs, Pathology and Genetics) as follows: 1) uterine carcinomas, including endometrioid adenocarcinoma (codes 8140, 8262, 8380, 8381, 8382, 8383, 8480, 8481, and 8570), serous adenocarcinoma (code 8461), clear cell adenocarcinoma (code 8310), and carcinosarcoma (codes 8950 and 8980); and 2) uterine sarcomas, including low grade endometrial stromal sarcoma (LGESS, code 8931), endometrial stromal sarcoma (ESS, code 8930), leiomyosarcoma (LMS, codes 8890, 8891, and 8896 ), and adenosarcoma (code 8933) [16].

### Statistical Analysis

The results were analyzed using Student’s t-test for pair-wise comparisons followed by one-way ANOVA for multiple group comparisons. Age-adjusted incidence rates (all females of any age) were calculated using the direct standardization method and the world population in 2000 as the standard population. The follow-up of each subject (in person-years) was calculated from the date of diagnosis to the date of death, or last date of linked data available from the Cancer Registry or Death Certification Profile, whichever came first, until December 31, 2010. Trends in age-specific incidence rates of endometrioid adenocarcinoma were subdivided by calendar year and birth cohort. Trends in age-specific incidence rates of type II uterine carcinomas (including serous adenocarcinoma, clear cell carcinoma, and carcinosarcoma) were subdivided by calendar year. The 5-year observed survival curves for women with uterine carcinomas and uterine sarcomas were calculated from 1990–2008 for each histological subtype using the National Cancer Registry. Survival curves by histological subtype were estimated using the Kaplan-Meier method, and differences between survival curves were assessed using the log-rank test. Cases lost to follow-up and those alive at the end of the follow-up period (December 31, 2010) were considered censored observations. A Cox-proportional hazards model was used to compare the 5-year uterine carcinoma and 5-year uterine sarcoma survival rates by histological subtype with adjustments for other risk factors, including age at diagnosis and period of diagnosis. The 95% confidence intervals (CI) for the hazard ratios (HR) were also calculated. Statistical significance levels were determined by two-tailed tests, and a p value less than 0.05 was considered statistically significant. Statistical analysis was performed with SAS software version 9.1 (SAS Inc, Cary, NC, USA).

## Results

### Basic Characteristics of Women with Uterine Cancer

**Figure 1:**
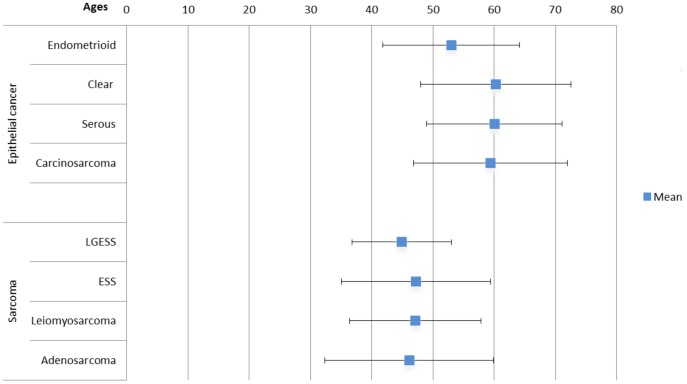
Mean ages of patients with uterine cancers by different histologic subtypes in Taiwan, 1979–2008 (mean ±1.96 S.D.).

Between January 1, 1979, and December 31, 2008, 13,839 women were diagnosed with uterine cancer. Their basic characteristics are shown in **Table 1**. The 83.5% of patients were uterine carcinomas, and 8.9% of cases were uterine sarcomas. The major histologic types of carcinomas and sarcomas were endometrioid adenocarcinoma (n = 10,546; 91.2%) and LMS (n = 715; 58.3%). As shown in **Table 1**, 339 women were diagnosed between 1979 and 1983, and gradually, larger numbers of individuals were diagnosed from 1984–1988 to 2004–2008, in both carcinomas and sarcoma. The peak incidence of age for uterine carcinomas was 50 to 59 years old. However, the peak incidence of age for uterine sarcomas was 40 to 49 years old, which was statistically significantly younger than for uterine carcinomas (*p*<0.0001, *t*-test). The mean ages of different histologic subtypes of uterine cancer are shown in **Figure 1**. The mean age of women with endometrioid adenocarcinoma (53.0 years) was younger than that of women with clear cell carcinoma (60.3 years), uterine serous carcinoma (60.1 years), and carcinosarcoma (59.5 years) (*p*<0.0001, one-way ANOVA test). In women with uterine sarcoma, the mean ages of each histologic subtype were 44.9 years for LGESS, 47.2 years for ESS, 47.2 years for LMS, and 46.1 years for adenosarcoma (*p = *0.17, one-way ANOVA test).

**Table 1:** Baseline characteristics of women with uterine cancer in Taiwan, 1979–2008 (n = 13,839).

Variable	Group	People(n = 13,839)	Carcinoma(n = 11,558)	Sarcoma(n = 1,226)
Period				
	1979–1983	339 (2.4%)	269 (2.3%)	5 (0.4%)
	1984–1988	706 (5.1%)	560 (4.8%)	56 (4.6%)
	1989–1993	1124 (8.1%)	888 (7.7%)	90 (7.3%)
	1994–1998	1886 (13.6%)	1497 (12.9%)	206 (16.8%)
	1999–2003	3235 (23.4%)	2680 (23.2%)	326 (26.6%)
	2004–2008	6549 (47.3%)	5664 (49.0%)	543 (44.3%)
Age (years)				
	<30	328 (2.4%)	171 (1.5%)	55 (3.8%)
	30–39	1339 (9.7%)	970 (8.6%)	231 (14.5%)
	40–49	3647 (26.4%)	2945 (25.9%)	506 (33.9%)
	50–59	4828 (34.9%)	4246 (36.9%)	282 (25.6%)
	60–69	2330 (16.8%)	2072 (17.6%)	98 (12.7%)
	>70	1235 (8.9%)	1062 (8.7%)	46 (8.7%)
	Unknown	132 (0.9%)	92 (1.8%)	8 (0.7%)
Subtype				
Carcinoma		11,558 (83.5%)		
	Endometrioid		10546 (91.2%)	–
	Clear		221 (1.9%)	–
	Serous		337 (2.9%)	–
	Carcinosarcoma		454 (3.9%)	–
Sarcoma		1,226 (8.9%)		
	ESS		–	321 (26.2%)
	LGESS		–	136 (11.1%)
	LMS		–	715 (58.3%)
	Adenosarcoma		–	54 (4.4%)
Others		1,055 (7.6%)		

ESS: endometrial stromal sarcoma, LGESS: low grade endometrial stromal sarcoma, LMS: leiomyosarcoma.

Our results indicated that patients with type II endometrial carcinomas (clear cell carcinoma, serous carcinoma, and carcinosarcoma) had a more advanced age in comparison with type I endometrial cancer (endometrioid adenocarcinoma). However, there were no significant differences in mean ages of uterine sarcomas between each histologic subtype.

### Compare the Incidence Rates of Uterine Cancer with Female Nasopharyngeal Carcinoma

**Figure 2:**
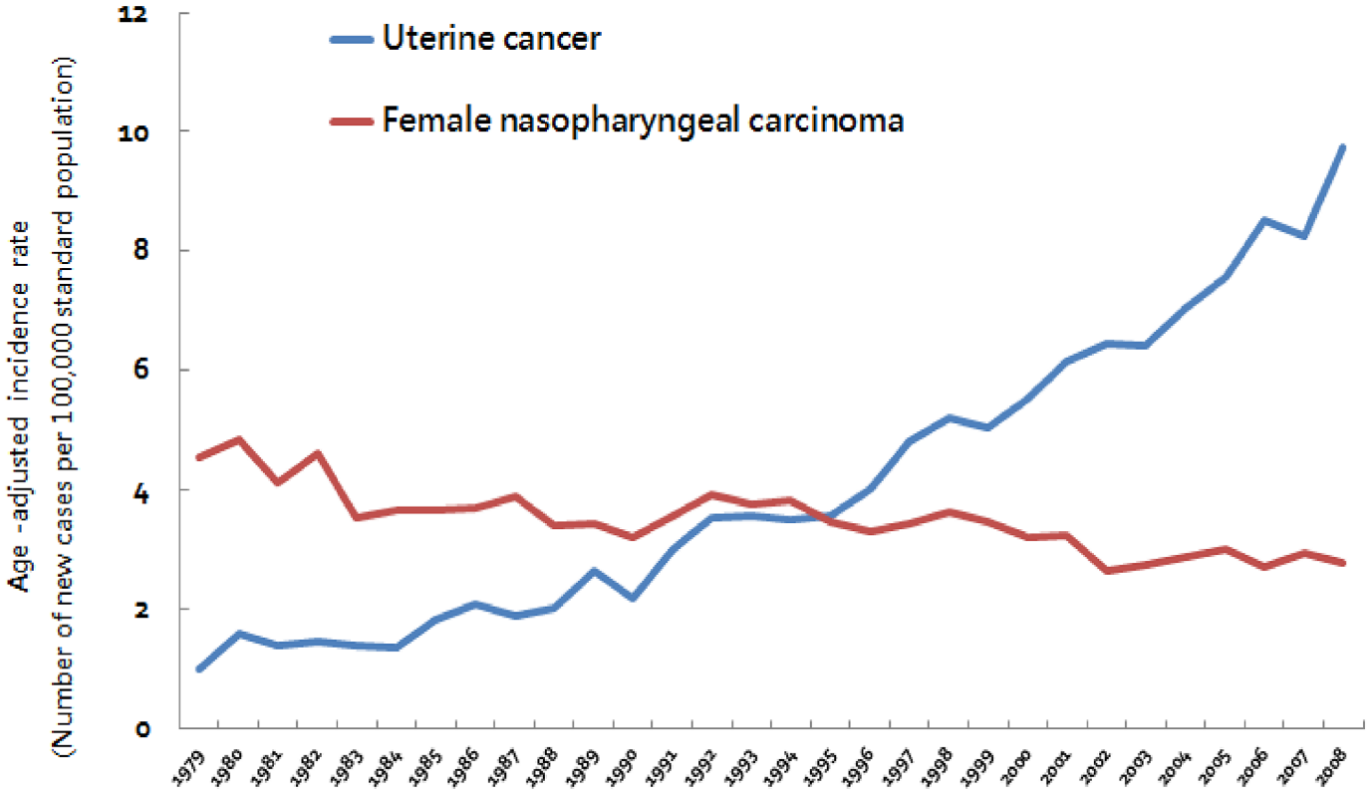
Secular trend of age-adjusted incidence rates of uterine cancer and female nasopharyngeal carcinoma, 1979–2008.

In uterine cancer patients, the overall increase in incidence and the changes between 5-year periods are exceptionally large. To avoid or reduce potential bias due to incomplete cancer registry data at the beginning of implementing the registry, we compared the age-adjusted incidence rates of uterine cancer with female nasopharyngeal carcinoma over the same period of time. As shown in **Figure 2**, from 1979 to 2008, the age-adjusted incidence rate of uterine cancer increased rapidly (0.99 per 100,000 women per year in 1979 and 9.75 per 100,000 women per year in 2008). In contrast, the incidence female nasopharyngeal carcinoma did not show a similar increase during the period of observation (4.55 per 100,000 women per year in 1979 and 2.77 per 100,000 women per year in 2008), according to the same registration system.

### Age-specific Incidence Rates of Endometrioid Adenocarcinoma by Calendar Year and Birth Cohort

**Figure 3:**
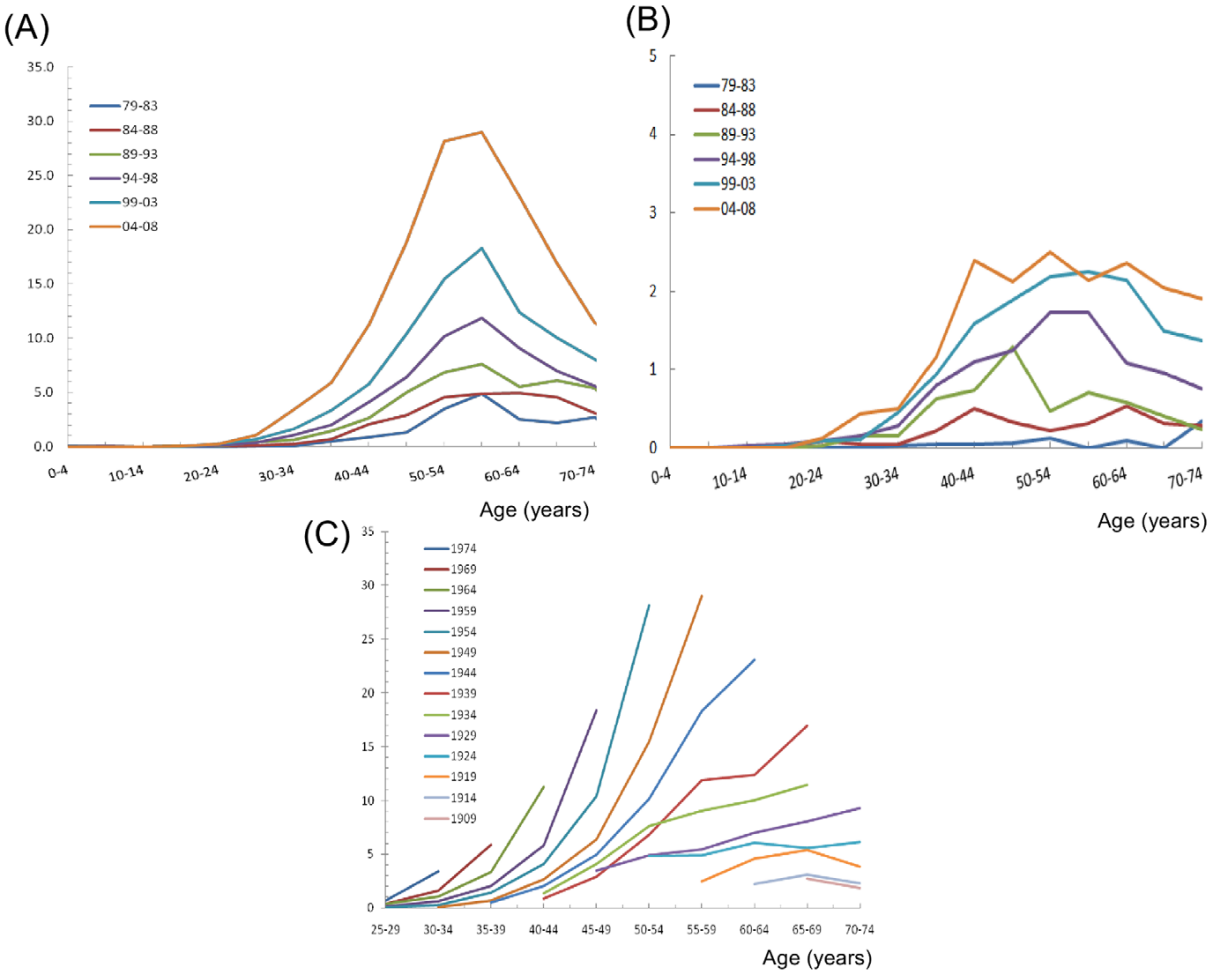
(A) Age-specific incidence rates of endometrioid adenocarcinomas by calendar year. (B) Age-specific incidence rates of type II uterine carcinomas by calendar year. (C) Age-specific incidence rates of endometrioid adenocarcinomas by birth cohort.

The age-specific incidence rates of endometrioid adenocarcinoma by calendar year are shown in **Figure 3A**. All of the age-specific incidence rates of endometrioid adenocarcinoma in different birth cohorts revealed bell-shaped curves with peak incidences at approximately 50 to 60 years of age. In addition, the age-adjusted incidence rate of endometrioid adenocarcinoma increased dramatically from 0.83 per 100,000 women per year (1979–1983) to 7.50 per 100,000 women per year (2004–2008). However, during the same period form 1979–2008, the age-specific incidence rate of type II endometrial carcinomas by calendar year (**Figure 3B**) had no significant change.

The birth cohort age-specific incidences of endometrioid adenocarcinoma are shown in **Figure 3C**. The incidence of endometrioid adenocarcinoma revealed an increasing trend in cohorts born after 1929. The slopes of the incidence of endometrioid adenocarcinoma for women born after 1944 were increasingly steeper than those for women born before 1944. In addition, the risk of endometrioid adenocarcinoma also significantly increased in women younger than 45 years old for those born after 1944.

Our results indicated that the birth cohort age-specific incidence of endometrioid adenocarcinoma increased more and more quickly in women with a younger age, especially for those born after the 1940s.

### Analyses of Long-term Survival of Uterine Carcinoma Patients

**Figure 4:**
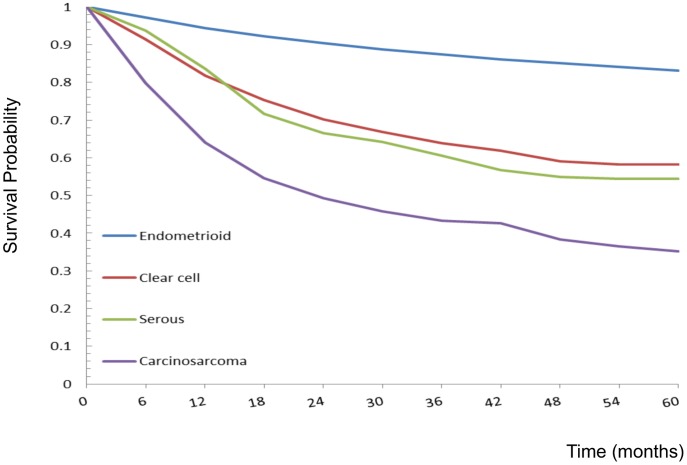
Five-year observed survival rates of uterine carcinomas by different histologic types: 1990–2008. Log-rank test X2 = 1105.72, p<0.0001.

There were a total of 10,365 patients with uterine carcinomas, with 54,665.3 person-years of follow-up and an average follow-up period of 5.3 years. As shown in **Figure 4**, the observed 5-year survival rates of uterine carcinomas were 83.2% (95% CI = 82.4–84.0%) among endometrioid adenocarcinomas, 58.3% (95% CI = 51.2–65.5%) among clear cell carcinomas, 54.4% (95% CI = 48.5–60.3%) among serous carcinomas, and 35.2% (95% CI 30.1–40.2%) among carcinosarcomas. Significant differences in the survival curves were observed across these four different histologic subtypes (log-rank X^2^ = 1105.72, *p*<0.0001), with the 20th percentile of the survival function (based on the failure probability) being higher for endometrioid adenocarcinoma (90 months) than for clear cell carcinoma (14 months), serous carcinoma (13 months), and carcinosarcoma (6 months).

Multivariate Cox proportional hazards analyses of overall survival in uterine carcinoma are shown in **Table 2**. When compared with women aged 40–49 years, those older than 50 years had significantly higher mortality risks (HR 1.43 for 50–59 years; HR 2.67 for 60–69 years; HR 4.54 for >70 years). The treatment period was also a significant factor for mortality risk. Compared with women treated from 1995 to 1999, the women treated after 2000 had a lower mortality risk (HR 0.80, 95% CI 0.71–0.91, *p*<0.001). In addition, the histologic subtype of uterine carcinoma was also an independent prognostic factor. The risks of mortality were significantly higher in the clear cell carcinoma, serous carcinoma, and carcinosarcoma groups compared with the endometrioid adenocarcinoma group (HR 2.05, 95% CI 1.65–2.55, *p*<0.001 for clear cell carcinoma; HR 2.55, 95% CI 2.15–3.03, *p*<0.001 for serous carcinoma; HR 4.60, 95% CI 4.03–5.25, *p*<0.001 for carcinosarcoma).

Our results indicated that age, treatment period, and histologic type were independent prognostic factors in women with uterine carcinomas.

**Table 2:** Multivariate Cox proportional hazards analysis of overall survival of patients with uterine carcinoma, 1990–2008 (n = 10,365).

Variable	Group	People	Person-years	Deaths	HR	95% CI	p
Age (years)							
	<30	147	853.6	21	1.13	0.73–1.76	0.58
	30–39	888	5398.3	98	0.91	0.72–1.13	0.39
	40–49	2712	15740.4	328	1	Referent	
	50–59	3813	19911.0	641	1.43	1.26–1.64	<0.001
	60–69	1847	9036.4	597	2.67	2.33–3.05	<0.001
	≥ 70	958	3725.6	480	4.54	3.93–5.24	<0.001
Period							
	1990–1994	1067	8478.1	324	0.95	0.83–1.09	0.49
	1995–1999	1877	15176.4	534	1	Referent	
	2000–2008	7421	31010.8	1,307	0.80	0.71–0.91	<0.001
Subtype							
	Endometrioid	9420	51385.5	1664	1	Referent	
	Clear	209	863.7	86	2.05	1.65–2.55	<0.001
	Serous	305	1239.9	145	2.55	2.15–3.03	<0.001
	Carcinosarcoma	431	1176.3	270	4.60	4.03–5.25	<0.001

HR: hazard ratio, CI: confidence interval.

### Analyses of Long-term Survival of Uterine Sarcoma Patients

**Figure 5:**
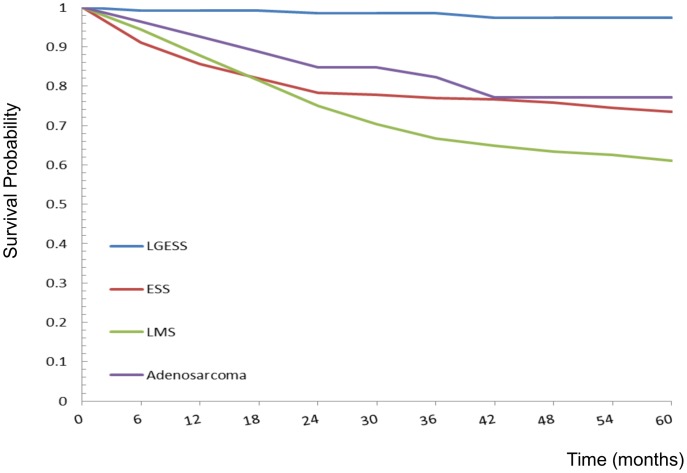
Five-year observed survival rates of uterine sarcomas by different histologic types: 1990–2008. Log-rank test X2 = 58.15, p<0.0001.

For the 1,137 patients with uterine sarcoma, there was a follow-up period of 5,917.2 person-years, with an average follow-up period of 5.2 years. The observed 5-year survival rate was 97.5% (95% CI 94.5–100.3%) among LGESS, 73.5% (95% CI 68.2.78.8%) among ESS, 60.1% (95% CI 57.1–64.9%) among LMS, and 77.2% (95% CI 65.1–89.2%) among adenosarcoma **(** **Figure 5** **)**. Significant differences in the survival curves were observed across different histologic subtypes (log-rank X^2^ = 58.15, *p*<0.0001). Multivariate analyses by Cox proportional hazards model are also shown in **Table 3**. When compared with women aged 40–49 years, those in older age groups (>50 years) had significantly higher risks of mortality (HR 2.53 for 50–59 years of age; HR 3.53 for 60–69 years; HR 3.30 for >70 years). The treatment period was another significant factor for the risk of mortality. As compared with women treated between 1995 and 1999, the women treated after 2000 had a higher risk of mortality (HR 1.52, 95% CI 1.08–2.14, *p* = 0.015). In addition, the histologic subtype of uterine sarcoma was also an independent prognostic factor. The risks of mortality were significantly higher in the LMS group compared with the ESS group (HR 1.59, 95% CI 1.24–2.04, *p*<0.001). In contrast, the risk of mortality was lower in the LGESS group compared with the ESS group (HR 0.13, 95% CI 0.05–0.32, *p*<0.001).

**Table 3:** Multivariate Cox proportional hazards analysis of overall survival of patients with uterine sarcoma, 1990–2008 (n = 1,137).

Variable	Group	People	Person-years	Deaths	HR	95% CI	p
Age(years)							
	<30	48	305.2	10	0.84	0.44–1.60	0.60
	30–39	215	1317.8	47	0.90	0.64–1.27	0.54
	40–49	482	2718.4	111	1	Referent	
	50–59	265	1097.3	132	2.55	1.98–3.28	<0.001
	60–69	86	334.8	51	3.53	2.52–4.93	<0.001
	≥ 70	41	143.8	24	3.30	2.11–5.15	<0.001
Period							
	1990–1994	135	1067.2	41	1.27	0.88–1.83	0.20
	1995–1999	255	1833.4	100	1	Referent	
	2000–2008	747	3016.6	234	1.52	1.08–2.14	0.015
Subtype							
	ESS	278	1716.9	84	1	referent	
	LGESS	135	624.2	5	0.13	0.05–0.32	<0.001
	LMS	670	3310.1	272	1.59	1.24–2.04	<0.001
	Adenosarcoma	54	266.0	14	0.83	0.47–1.46	0.52

HR: hazard ratio, CI: confidence interval, ESS: endometrial stromal sarcoma, LGESS: low grade endometrial stromal sarcoma, LMS: leiomyosarcoma.

Our results indicated that age, treatment period, and histologic types were also independent prognostic factors in women with uterine sarcomas.

## Discussion

The data from this nationwide, population-based study provided several important findings. First, the number of cases of hormone-dependent type I endometrial cancer has increased rapidly in the past 30 years in Taiwan compared to other countries. Second, patients with clear cell carcinoma, serous carcinoma, and carcinosarcoma had worse outcomes than those with endometrioid adenocarcinoma. Third, the incidences of endometrioid adenocarcinoma increased more and more quickly in women with a younger age, especially for those born in recent cohorts. Fourth, differences in the treatment period affected the outcome of women with uterine cancers, regardless of whether they had carcinomas or sarcomas.

In the current study, an increase over time of type I endometrial cancer among Taiwanese women during the past 30 years was identified rather than type II endometrial cancer. However, this situation has not been observed in Caucasians in the United States [17]. The incidence rates of endometrial cancer has also increased in western European countries [18] and other Asian countries such as Japan and China, but with a relatively slower increase rate compared to that in Taiwan. The age-standardized rate (based on 1996 world standard population) of uterine cancer was 2.6 per 100,000 women per year in 1983 and 4.0 per 100,000 women per year/in 2002 in Japan, and 4.6 per 100,000 women per year in 1988 and 8.2 per 100,000 women per year in 2002 in China [19]. However, the age-adjusted incidence rates of endometrioid adenocarcinoma (based on 2000 world standard population) were 0.83 per 100,000 women per year/from 1979 to 1983 and 7.50 per 100,000 women per year from 2004 to 2008 in Taiwan in this survey. The same trend in hormone-related breast cancer among Taiwanese women compared to women in the United States was also observed in our previous study [20]. The role of unopposed or excess estrogen exposure in the development of type I endometrial cancer has been clearly established [21], [22]. Excess estrogen may come from either endogenous or exogenous sources. Taiwan underwent rapid industrialization in the 1960s, and Taiwanese women born after the 1960s tend have an earlier menarche, delayed childbearing and reduced fertility rates [23]–[25]. These risk factors can lead to prolonged, unopposed endogenous estrogen stimulation of the endometrium [26]. In addition, the body mass index of Taiwanese women has increased in past 30 years due to a westernization of lifestyles [27]. Obesity also can cause a hyperestrogenic state through an increased aromatization of estrogen precursors in fatty tissues, and this may be the main mechanism linking obesity with the risk of endometrial cancer [28].

In addition to endogenous estrogen, exogenous pollutants with estrogenic effects may be another possible factor for the increasing incidence of endometrial adenocarcinoma in Taiwanese women. Phthalates are widely used in industry and consumer products in Taiwan, and di-(2-ethylhexyl) phthalate (DEHP) and di-*n*-butylphthalate (DBP) have been demonstrated to be the most potent reproductive toxicants among the phthalates. Chen and colleagues [29] reported that the intensive use of plastics in food handling has resulted in high exposure to DEHP in Taiwan. In addition, high concentrations of polycyclic aromatic hydrocarbons in the air and estrogenic steroid pollutants in water have been also reported in Taiwan [30], [31]. All of these exogenous pollutants may contribute to the exogenous estrogen effect resulting in the increasing number of cases of type I endometrial cancer.

To reduce potential bias due to incomplete cancer registry data (less complete reporting, less insurance coverage, or un-experienced doctors/staff in early years of the registry), the incidences of female nasopharyngeal carcinoma over the same period of time was selected as reference controls. The incidence rate of female nasopharyngeal carcinoma was relatively stable during the period of 1981–2007 in Taiwan, but the incidence of uterine cancers was found to increase to a much greater extent than that of the reference controls. So the possibility that increased incidence rate of uterine cancer was affected by incomplete cancer registry data at the beginning of implementing the registry should be less important.

Histologic subtype was an independent prognostic factor for uterine carcinoma and sarcoma. We found that women with clear cell carcinoma, serous carcinoma, and carcinosarcoma had poorer outcomes than those with endometrioid adenocarcinoma. Hamilton et al. also found that tumor histology was an important prognostic factor in patients with uterine carcinoma in a population-based study [32]. Carcinosarcomas (malignant mixed mesodermal tumors or MMMT) are currently classified as metaplastic carcinomas. The behavior of uterine carcinosarcoma is aggressive and similar to that of high-grade endometrioid type endometrial adenocarcinoma and aggressive morphological subtypes of uterine carcinoma. The adjuvant treatments should probably be similar to those directed against aggressive high grade endometrial carcinomas [9]. We then classified carcinosarcoma as uterine carcinoma and compared its outcome with other subtypes of uterine carcinomas and found that carcinosarcoma has the worst outcome with a 35.2% 5-year survival rate. Women with LGESS had the best outcomes compared with other histologic types in uterine sarcomas in our survey. There were only five deaths observed among 135 women with LGESS with a 5-year survival rate of 97.5% in this study. Piver et al. also demonstrated a prolonged survival of LGESS patients, with a 10-year survival rate of 89% [33].

The limitation of this study is a lack of cancer staging, which could be a prognostic factor for the outcomes of uterine cancer patients. Previous investigations have demonstrated that tumor stage is a prognostic factor in both uterine carcinomas and sarcomas [34]–[36]. The National Cancer Registry of Taiwan, which we used for analysis, lacks information regarding tumor stage. In contrast to endometrioid adenocarcinoma, most of the patients with clear cell carcinoma and serous carcinoma are diagnosed at advanced stages with disease spread beyond the uterus [8], [37], [38]. We believe that stage was also an independent prognostic factor in this survey; however, the National Cancer Registry of Taiwan did not include information on cancer staging until 2007. Despite this limitation, our study still provides a large-scale, nationwide, population-based study regarding the incidence and long term survival status of patients with uterine cancers without potential selection biases.

Patients with uterine carcinomas diagnosed and treated after 2000 had better outcomes. The treatment of uterine carcinomas has changed in the past 10 years, and ever increasing numbers of patients undergo comprehensive surgical staging operations including extrafascial hysterectomy, bilateral salpingo-oophorectomy, and pelvic and para-aortic lymphadenectomy. Selective pelvic and para-aortic lymphadenectomy has been suggested to have a therapeutic effect with survival benefits [39], [40]. Most cases of uterine cancer have been treated by gynecologic oncologists in Taiwan since 2000, but this was not the case before 2000. Chan et al. showed that patients treated by gynecologic oncologists undergo more comprehensive staging surgery and adjuvant chemotherapy for advanced disease in United States [41]. Care provided by gynecologic oncologists also improved the survival of the patients with high-risk cancers [41]. In addition, the availability of easily applied diagnostic tools [42] and a clearer understanding of premalignant lesions of the endometrium have led to an increase in the number of women diagnosed with endometrial cancer with better outcomes. Taken together, these findings can explain the survival differences of patients with uterine adenocarcinoma in this survey.

In contrast, women with uterine sarcomas diagnosed and treated after 2000 had less favorable outcomes in this survey. After further analysis, we found that the poorer outcomes were mainly in women younger than 40 years old with leiomyosarcoma rather than older women and other histologic types of sarcomas (data not shown). Because most patients with uterine leiomyosarcoma were treated as benign uterine fibroid before surgery, laparoscopic operations such as laparoscopic myomectomy or hysterectomy without complete staging surgery were always performed for these women. In addition, Park et al. reported that tumor morcellation during surgery increased the rate of abdomino-pelvic dissemination and adversely affected disease-free survival and overall survival in patients with uterine leiomyosarcoma [43]. These findings may contribute to our observations that women with uterine sarcomas treated after 2000 had poorer outcomes.

The strengths of the current study include the nationwide, population-based study design including almost all incident uterine cancer patients using the National Cancer Registry of Taiwan, as well as the long-term follow-up of survival status for all uterine cancer patients by death certification, resulting in a large, reliable study cohort and robust results without potential selection biases. The major shortcoming of this study is the lack of information regarding cancer staging which may have influenced the interpretation of the data. Another limitation of our study is a lack of central pathology. The discordance between different pathologists with regards to either site of origin or tumor histopathologic type may also have influenced the study results. In addition, the average follow-up for survival was relatively short. The average follow-up for survival was 5.3 years in patients with uterine carcinomas and 5.2 years in patients with uterine sarcomas. However, based on this nationwide, population-based study, the large number of patients in this series may have overcome these potential limitations. We believe that our findings still provide meaningful insights into the study of uterine cancer patients which in turn warrant further studies to investigate the possible underlying mechanisms.
